# Pemigatinib-Associated Psoriasis in a Patient With Cholangiocarcinoma

**DOI:** 10.7759/cureus.108439

**Published:** 2026-05-07

**Authors:** Abigail G Hendrie, Lina Hadj Smaine

**Affiliations:** 1 David Geffen School of Medicine, University of California, Los Angeles, Los Angeles, USA; 2 Department of Medicine, University of California, Los Angeles, Los Angeles, USA

**Keywords:** cholangiocarcinoma, drug-induced psoriasis, fgfr inhibitor, pemigatinib, psoriasis

## Abstract

Psoriasis is a chronic autoimmune skin disease that may be triggered or exacerbated by medications, including targeted oncologic therapies. Pemigatinib is a fibroblast growth factor receptor inhibitor used for the treatment of cholangiocarcinoma. We present the case of a 61-year-old woman with unresectable cholangiocarcinoma and lung metastases who developed psoriatic plaques after four cycles of pemigatinib. She had a remote history of limited psoriasis but no other new triggers. Her presentation included occipital scalp and leg plaques. Topical therapies were prescribed; however, follow-up revealed persistent disease and uncertain treatment adherence. The patient ultimately deprioritized dermatologic symptoms in the context of systemic illness. To our knowledge, based on searches in PubMed and other databases, this represents the first reported case of pemigatinib-induced psoriasis. Recognition and management of dermatologic adverse effects are critical to minimizing morbidity in the setting of life-prolonging oncologic treatments. Given the limitations of a single-case inference, further surveillance and study are needed to determine whether patients with pre-existing psoriasis may be at increased risk.

## Introduction

Psoriasis is a chronic autoimmune inflammatory condition that presents with well-defined erythematous plaques with silvery scales, commonly involving the scalp, elbows, knees, and presacral region [1]. While psoriasis may arise idiopathically or in association with well-established triggers, psoriasis associated with fibroblast growth factor receptor (FGFR) inhibitors has not previously been reported. Here, we describe the first known case of pemigatinib-associated psoriasis, highlighting a potential novel dermatologic adverse effect of this targeted therapy.

Psoriasis is a common disease, with a prevalence ranging from 0.2% to 4.8%. The condition is thought to be driven by T lymphocytes, which stimulate the proliferation of keratinocytes, resulting in the formation of thick plaques. Other related characteristics include thickening of the epidermis and parakeratosis. The epidermal cells do not properly secrete lipids, leading to the flaky, scaly skin commonly seen in psoriasis. Many patients with psoriasis demonstrate links to certain human leukocyte antigens, especially across different racial and ethnic populations. Its occurrence within families also suggests a genetic susceptibility [2].

Triggers include infections, stress, cutaneous trauma, smoking, alcohol consumption, and medications such as beta-blockers, lithium, antimalarials, and nonsteroidal anti-inflammatory drugs [3,4]. Monoclonal antibody- and small-molecule-based targeted therapies for oncologic and immunologic diseases have more recently been implicated, such as tumor necrosis factor-alpha antagonists and anti-programmed cell death protein 1 immune checkpoint inhibitors. Drug-induced psoriasis typically presents similarly to nondrug-related forms, thus presenting a challenge for clinical distinction. Additionally, the period between initiation of a drug and onset of psoriasis can be significantly long for some drugs [5].

The prognosis for cholangiocarcinoma has historically been poor, with chemotherapy as the only systemic option and overall survival under one year. Recent studies have identified key genetic drivers, including FGFR family mutations. FGFR tyrosine kinases are membrane-bound proteins that play key roles in numerous cellular processes, including proliferation, survival, migration, and differentiation [6]. The discovery of FGFR gene fusions and other alterations across various solid tumors, including cholangiocarcinoma, has driven the development of targeted FGFR inhibitors for therapeutic use. The FGFR inhibitor pemigatinib was approved by the U.S. FDA in April 2020 for previously treated patients with FGFR2 rearrangements, introducing targeted therapy for this disease [7].

Pemigatinib is a kinase inhibitor with antitumor activity. It is a small-molecule inhibitor of FGFR1, FGFR2, and FGFR3. Normally in the skin, FGFRs suppress inflammation by reducing the expression of inflammatory cytokines and interferon-stimulated genes. By blocking FGFR signaling, pemigatinib may lead to a proinflammatory state that could precipitate autoimmune and autoinflammatory conditions such as psoriasis in susceptible individuals [8]. FGFR inhibitors have known dermatologic adverse effects, including alopecia, palmar-plantar erythrodysesthesia syndrome, and calcinosis cutis/calciphylaxis. However, to our knowledge, this represents the first reported case of FGFR inhibitor-associated psoriasis.

## Case presentation

We present the case of a 61-year-old female with unresectable cholangiocarcinoma and lung metastases who developed a new rash after starting the FGFR inhibitor pemigatinib (13.5 mg daily, two weeks on/one week off). The patient started pemigatinib after failed cycles of gemcitabine and cisplatin. Reported side effects after two weeks included nausea and vomiting, blurry vision, body aches, headaches, and worsening foot neuropathy. After four cycles, she reported progressive scalp thickening and scaling, with itchiness rated 8/10 on the Numerical Rating Scale [9]. She endorsed a remote history of limited psoriasis on the neck for several years but denied recent infection, trauma, smoking, alcohol use, or other new medications. Hydrocortisone cream and coconut oil shampoo did not provide relief.

**Figure 1:**
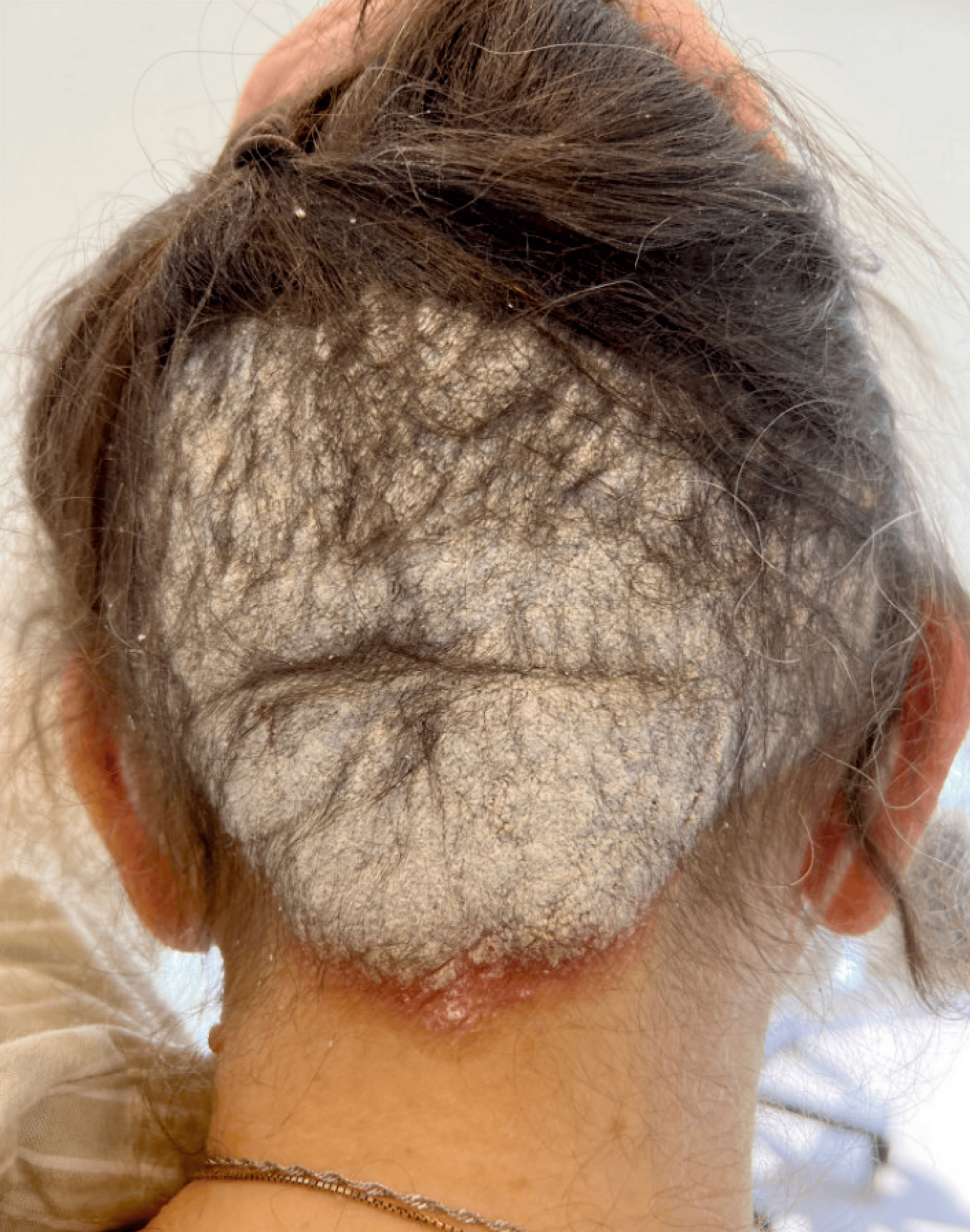
Clinical presentation of scalp psoriasis associated with pemigatinib therapy Initial presentation of a well-demarcated erythematous plaque with overlying thick, silvery scale involving the lower posterior scalp, consistent with psoriasis. The lesion demonstrates characteristic scaling and epidermal thickening typical of psoriatic involvement.

**Figure 2:**
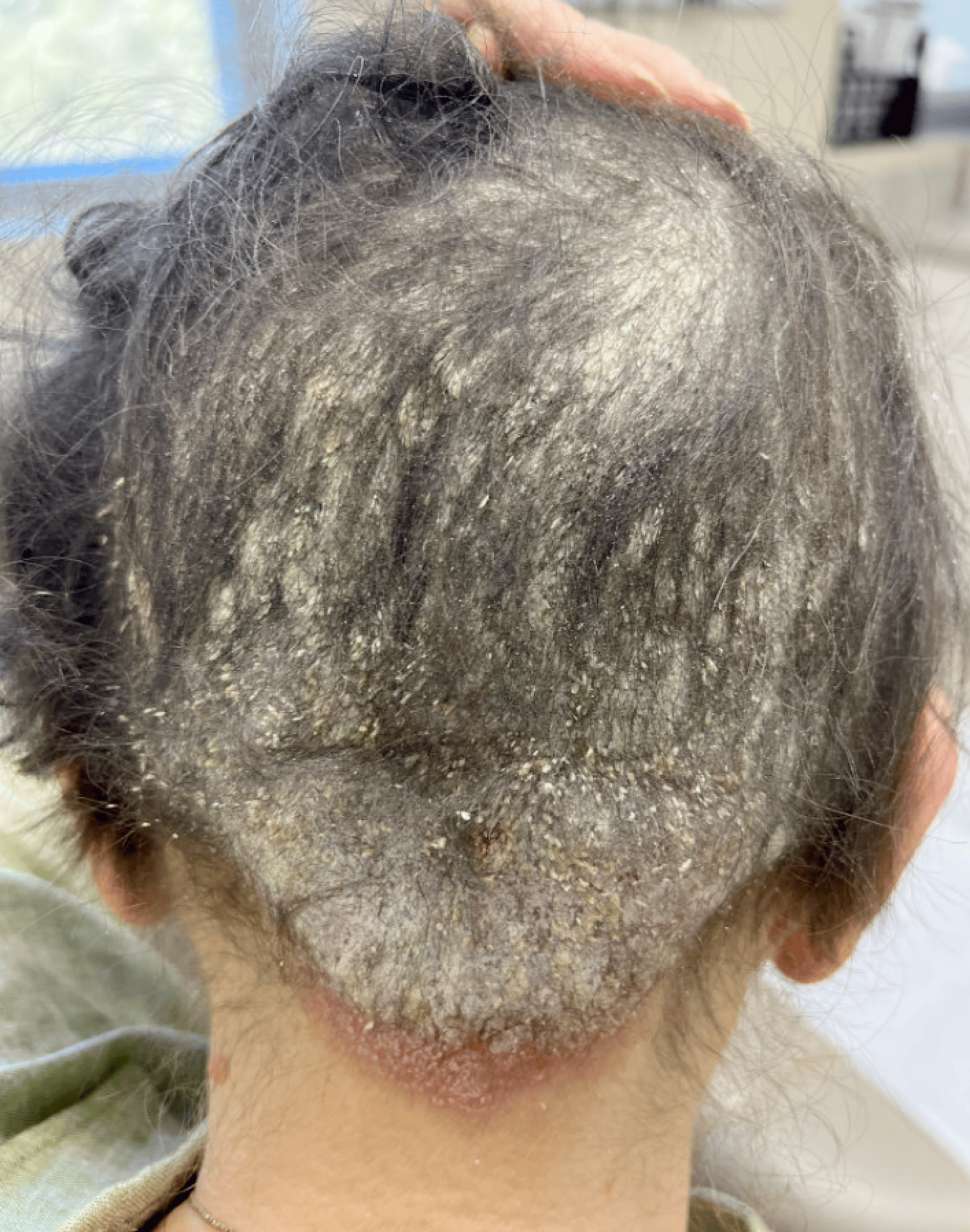
Posterior scalp psoriasis six days after initial presentation, prior to treatment Posterior scalp demonstrating a persistent, well-demarcated erythematous plaque with thick silvery scale, consistent with psoriasis.

On examination, she had an erythematous, flaking, well-demarcated plaque covering the mid-occipital scalp and smaller plaques on the anterior legs (Figure 1, Figure 2).

Severity was scored as 9-10% body surface area and Investigator’s Global Assessment 4, indicating severe disease [10]. Hydrocortisone was discontinued, and treatment with fluocinonide 0.05% solution and ketoconazole 2% shampoo was prescribed. She was, several months later, taken off pemigatinib due to disease progression. She was started on futibatinib, an FGFR1-4 inhibitor. On subsequent follow-up, the patient reported persistent scalp involvement and was uncertain whether she had used the prescribed topical treatment. When offered further dermatologic management, she expressed that the psoriasis was not a priority given her overall health concerns (Table 1).

**Table 1:** Timeline of clinical course BSA, body surface area; IGA, Investigator’s Global Assessment

Time point	Clinical events	Findings	Management
Baseline	Initiated pemigatinib for unresectable cholangiocarcinoma	No active psoriasis reported	-
Cycle 1 (weeks 0-2)	Initiated pemigatinib 13.5 mg daily (two weeks on/one week off)	Developed systemic side effects (nausea, blurry vision, neuropathy); no reported skin findings	Supportive care
Cycle 4 (month 3)	New scalp rash	Erythematous, well-demarcated plaque on the occipital scalp; smaller plaques on legs; BSA 9-10%, IGA 4	Hydrocortisone and coconut oil without relief; fluocinonide 0.05% and ketoconazole shampoo prescribed
Six months later	Discontinued pemigatinib due to disease progression; futibatinib started	Dermatologic status not the primary concern	Oncology-directed management
Follow-up several months later	-	Persistent dermatologic symptoms	Patient declined further dermatologic intervention

## Discussion

Pemigatinib is generally well tolerated from a dermatologic standpoint, although several cutaneous adverse effects have been reported. In clinical studies, alopecia is among the most common dermatologic toxicities, occurring in approximately 46% of treated patients, predominantly as grade 1-2 events. Palmar-plantar erythrodysesthesia syndrome has also been observed, with grade 3-4 severity reported in approximately 4% of patients. In addition, rare but potentially serious cutaneous complications have been described, including a reported case of calcinosis cutis/calciphylaxis in a patient receiving pemigatinib [6]. Other known side effects of pemigatinib include ocular toxicity, hyperphosphatemia, soft tissue mineralization, and alopecia (Table 2) [11].

**Table 2:** Adverse effects of pemigatinib and their treatments Source: [11]

Adverse effect	Treatment
Ocular toxicity	Artificial tears, lubricating eye gels
Hyperphosphatemia	Low-phosphate diet, phosphate binders
Soft tissue mineralization	Control of phosphate levels
Alopecia	Reassurance; regrowth typically begins soon after cessation of treatment

Dermatologic side effects, including alopecia and onycholysis, have been reported with the use of futibatinib, an FGFR inhibitor [12]. To our knowledge, this is the first reported case of pemigatinib-associated psoriasis, highlighting the need for awareness of this potential adverse effect. This patient’s rash is consistent with psoriasis, given its characteristic features and reported response to treatment. The patient also had a history of psoriasis, further supporting the diagnosis. The timing of the onset of psoriasis, a few months after initiation of pemigatinib, provides supportive evidence of the drug as the trigger for this patient’s flare.

FGFR plays a key role in the suppression of inflammation. When its signaling is blocked, patients may be in a state more susceptible to inflammatory diseases [8]. Several months of increased inflammation due to the lack of FGFR-mediated suppression caused by pemigatinib may create an immune environment vulnerable to flaring of autoimmune conditions, such as psoriasis.

The delay between initiation of therapy and onset of symptoms highlights the importance of provider awareness of this possible side effect. Other cases of drug-induced psoriasis have been documented with several other drug classes, with clinical improvement following initiation of therapy and/or discontinuation of the offending agent when possible. In our patient’s case, the potential benefit of treatment for cholangiocarcinoma outweighed the risks of adverse effects. Despite the availability of topical treatments, the patient experienced persistent symptoms and did not prioritize dermatologic care in the context of systemic illness. This underscores that real-world management of cutaneous toxicities may be influenced by competing health concerns, patient preferences, and treatment burden. Clinicians should remain aware of these adverse effects, even when they may not be the primary concern for patients.

To further assess causality, the Naranjo Adverse Drug Reaction Probability Scale was applied, yielding a score consistent with a probable adverse drug reaction [13]. This assessment is supported by the temporal relationship between pemigatinib initiation and onset of symptoms, the absence of alternative triggers, and the clinical examination. Limitations of this case include the lack of biopsy confirmation, which may reduce diagnostic certainty and reproducibility. The differential diagnosis included seborrheic dermatitis, tinea capitis, and other psoriasiform drug eruptions. However, the diagnosis of psoriasis was strongly supported by the clinical morphology, distribution, and history of psoriasis.

## Conclusions

To our knowledge, this represents the first reported case of pemigatinib-associated psoriasis, expanding the spectrum of dermatologic adverse effects associated with FGFR inhibition. Given the limitations of a single-case inference, further post-marketing surveillance will be essential to determine whether patients with pre-existing psoriasis are at increased risk and to better characterize the dermatologic safety profile of FGFR inhibitors. Clinicians should remain aware of possible inflammatory cutaneous reactions in patients receiving FGFR inhibitors, even when these symptoms may not be prioritized by patients in the context of serious systemic illness.
